# Bilateral Pleural Effusion in a Patient with an Extensive Extramedullary Hematopoietic Mass

**DOI:** 10.1155/2013/857610

**Published:** 2013-08-18

**Authors:** Yun Luo, Ying Zhang, Shi-feng Lou

**Affiliations:** Department of Hematology, Second Affiliated Hospital, Chongqing Medical University, No. 76, Linjiang Road, Chongqing 400010, China

## Abstract

We present a 56-year-old woman with bilateral pleural effusions, widespread enlarged lymph nodes, and soft tissue masses located within the renal pelvis. The initially working diagnosis was tuberculosis and lymphoma. Further pathological examination of the lymph node biopsy confirmed a diagnosis of extramedullary hematopoiesis, and a bone marrow biopsy revealed myelofibrosis. Unlike common treatment options such as radiotherapy and/or surgery, intrathoracic cisplatin and dexamethasone for the treatment of pleural effusions secondary to extramedullary hematopoiesis demonstrated an improvement in feasibility and efficacy in the present case.

## 1. Introduction

Extramedullary hematopoiesis (EMH) is the recurrence of normal marrow outside of the skeleton following birth. It is most commonly associated with thalassaemia, sickle cell anaemia, or hereditary spherocytosis and rarely with myelofibrosis. EMH develops predominantly in the liver and spleen; however, it may also occur at other sites including the thymus, central nervous system, lymph nodes, lung, pleura, myocardium, kidney, retroperitoneum, and paravertebral areas of the thorax [1]. We reported one case of EMH involved in widespread lymph nodes with bilateral pleural effusions which was treated successfully with an intrathoracic injection of cisplatin and dexamethasone, coupled with oral prednisone, hydroxyurea, and thalidomide treatment.

## 2. Case Report

A 56-year-old female was admitted to our hospital in November 2009 with a one month history of fatigue, cough, and dyspnea. Past medical history indicated a prior splenectomy 20 years ago due to splenomegaly, positive HBS-Ag for 10 years, and bone tuberculosis 4 years ago treated with antituberculosis drugs for one year. On physical examination, neck and inguinal lymph nodes could be touched, bilateral dullness and reduced breath sounds were found, and the liver was enlarged by three centimeters. 

Initial blood counts revealed a hemoglobin of 110 g/dL, hematocrit 34.3%, mean corpuscular volume 93.7 fL, white blood cell count 71.1 × 10^9^/L, and platelets 57 × 10^9^/L. Peripheral blood analyses showed a leukoerythroblastic picture (lymphocytes 10%, monocytes 1%, myelocytes 13%, metamyelocytes 4%, segmented neutrophils 18%, band neutrophils 15%, orthochromatic erythroblasts 35%, polychromatophilic erythroblast 3%, and eosinophils 1%) with poikilocytes, acanthocytes, conjugate nuclei erythrocytes, and target erythrocytes. Serum lactic dehydrogenase was 732 U/L (normal 50–245 U/L), *α*-hydroxybutyric acid 542.4 U/L (72–182 U/L), and C-reactive protein 13.17 mg/L (0–4 mg/L). Immunoglobulin, serum protein electrophoresis, ferritin, hemoglobin A2, and percentage fetal hemoglobin were within normal ranges. Pleural fluid was observed to be exudative (total protein 29.3 g/L, Rivalta's test positive) with a red blood cell count of 6.35 × 10^9^/L and white blood cell count of 6.9 × 10^9^/L (automated count: 5% neutrophils, 94% lymphocytes, and 1% eosinophils). No bacteria or tubercle bacillus were found within the pleural fluid.

**Figure 1:**
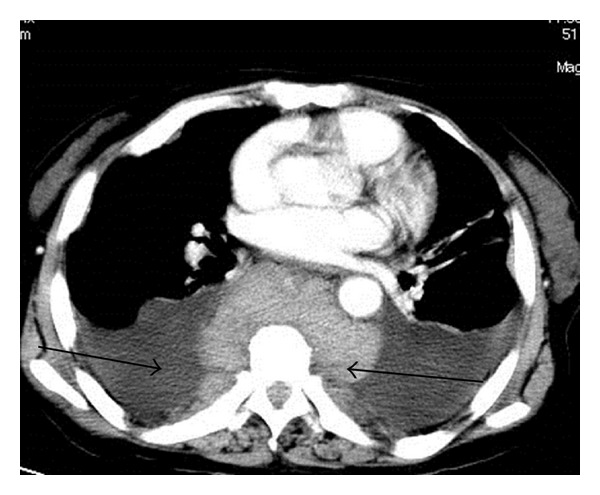
CT result suggests multiple round masses in the paraspinal and thoracic regions and bilateral pleural effusions. On November 13, 2009, CT scan was performed, and the left arrow indicated pleural effusion, and the right arrow suggested rounded soft mass surrounding vertebrae.

**Figure 2:**
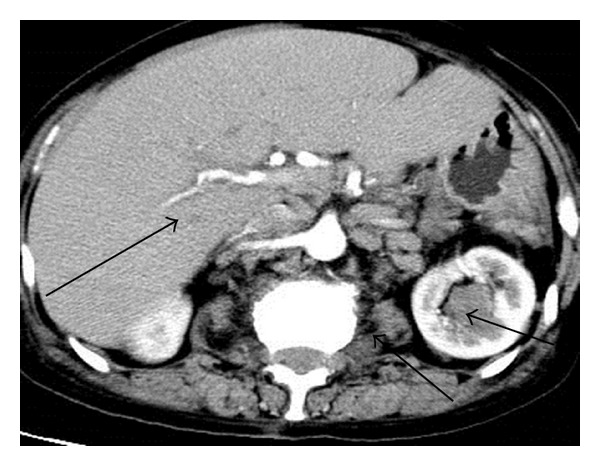
CT result shows multiple lymph nodes in the peritoneal cavity and retroperitoneal area. An enlarged liver and soft tissue masses were observed in the left kidney. On November 13, 2009, CT scan was performed, and the left arrow indicated enlarged liver with homogenous density, the middle arrow suggested enlarged lymph nodes in the abdomen, and the right arrow represented a soft tissue mass in the left kidney.

Computed tomography (CT) of the chest and abdomen revealed massive bilateral pleural effusions with pleural thickening and ground-glass appearance of the upper lung lobes. Slightly enlarged lymph nodes of mixed density were observed at the local thoracic paravertebral area within the posterior mediastinum and retroperitoneal paravertebral space which spread along the abdominal aorta and iliac artery. CT also identified hepatic enlargement with interstitial edema, loss of the corticomedullary differentiation of the left kidney, and soft tissue masses in the left renal pelvis (Figures 1 and 2). The radiologist suggested an impression of lymphoma. 

**Figure 3:**
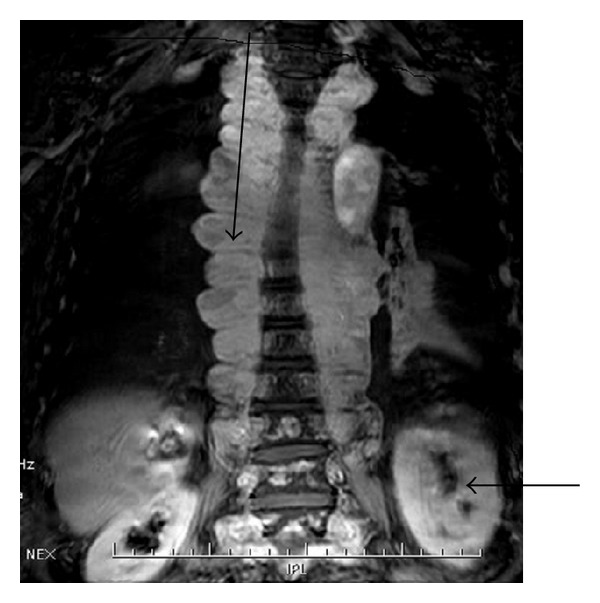
MRI shows asymmetrical soft tissue mass with a stringy appearance surrounding the thoracic vertebral bodies. On November 15, 2009, an MRI was performed. The upper arrow indicated soft tissue masses with a stringy appearance surrounding the thoracic vertebral bodies, and the lower arrow suggested a soft tissue mass in the left kidney.

Magnetic resonance imaging (MRI) of the thoracic waist spine was obtained using a 1.5 Tesla unit. T1-weighted images revealed punctuate nodular short signals located at the T3-L5 level. T2-weighted images showed a slightly long signal of patching hypointensity within the spinal cord. The vertebral bodies were surrounded by an asymmetric paravertebral asymmetrical soft tissue mass which appeared stringy in nature and was enhanced on T1W1 (Figure 3).

**Figure 4:**
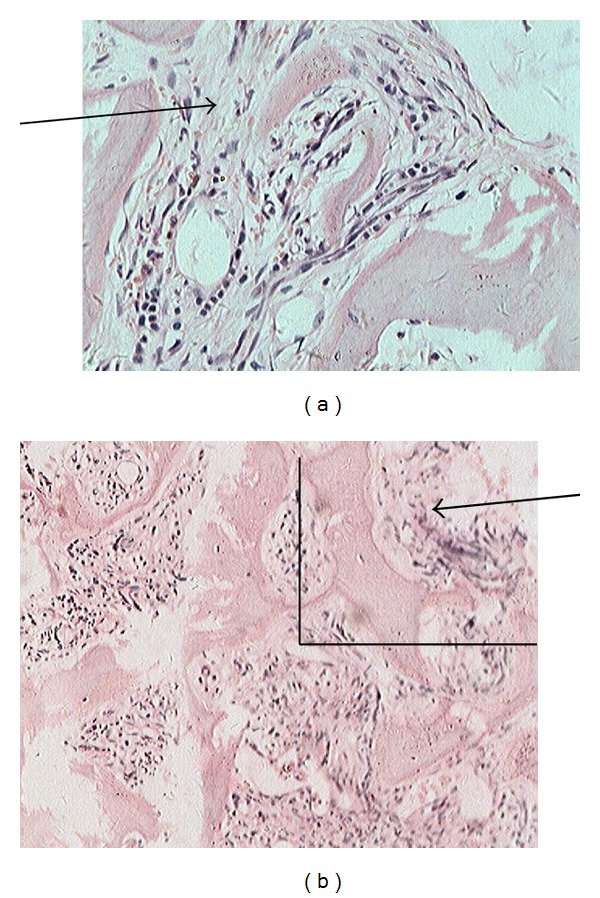
Iliac bone marrow biopsy shows increased fibroblasts and reduced marrow cellularity with an increase in reticulin (100 and 200x). On November 18, 2009, an iliac bone marrow biopsy was performed in the patient, and the slide was stained with H&E. The arrows show increased fibrous tissue. The upper panel (200x) is a blowup of the panel (100x) within the square area.

Results from an iliac bone marrow aspiration showed hypocellularity with some features of myelodysplasia including dikaryon and toxic granulation, but no ringed sideroblasts were observed (not shown). A bone marrow biopsy unveiled increased fibrous tissues and discrete marrow cellularity with increased reticulin staining (Figure 4). The bone marrow cells had found the JAK2V617F mutation; chromosome banding (Giemsa-trypsin) demonstrated a normal 46XX karyotype. Intestinal fiberscope and ultrasonography showed no evidence of a tumor in other organs and tissues.

**Figure 5:**
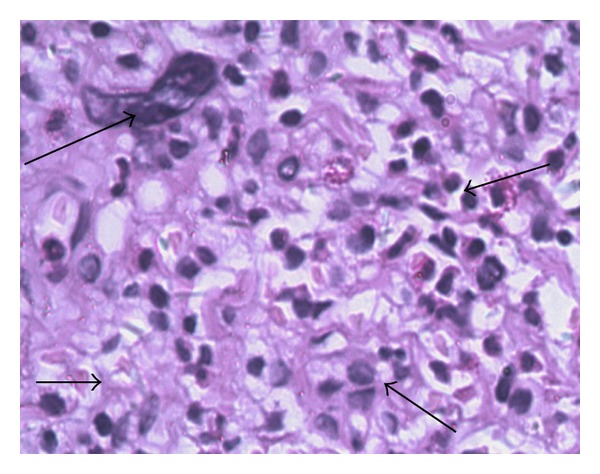
Lymph node biopsy reveals disorganization with myeloid and erythroid precursors among the fat. Several cells show karyokinesis (H&E staining 400x). On November 21, 2009, a lymph node biopsy was performed. The left upper arrow showed karyokinesis, the left lower arrow indicated widespread fat cells, the right upper arrow demonstrated erythroblasts, and the right lower arrow suggested myeloblast.

The patient underwent an inguinal lymph node biopsy, and the results revealed disorganized architecture with myeloid and erythroid precursors dispersed among adipose tissue and karyokinesis within the cells (Figure 5). The biopsy stained positive for CD3, CD20, CD43, CD45RO, CD79*α*, myeloperoxidase (MPO), Ki67, LCA, CD15, and P80; and it stained negative for CD30 and cytokeratin together with acid fast stain (not shown). Eventually, EMH was diagnosed.

**Figure 6:**
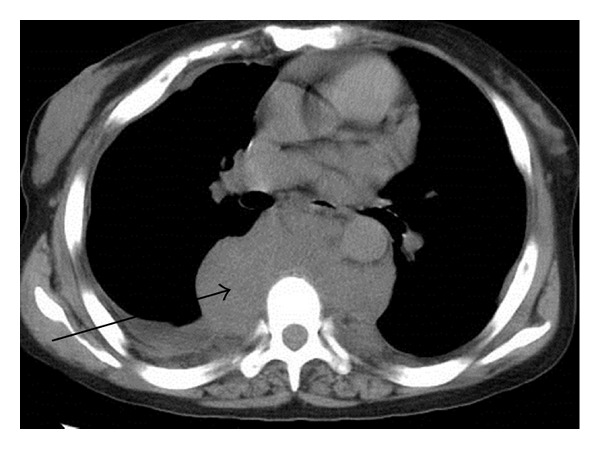
Post-treatment CT result suggests bilateral pleural effusions were largely not evident, however soft masses surrounding the vertebrae still exist. On March, 6, 2011, the CT scan was performed following treatment. The left arrow indicated a rounded soft tissue mass surrounding the vertebrae.

The patient was treated with oral prednisone 30 mg/qd, temporary diuretics, and hydroxyurea 1 g/qd for two weeks followed by continuing prednisone and thalidomide 50 mg/qn for up to six months. She also received an intrathoracic injection of dexamethasone 10 mg and cisplatin 30 mg. Her symptoms improved, and pleural effusion decreased. She also received intrathoracic injection of cisplatin again after 112 days due to increasing dyspnea and recurrence of the bilateral pleural effusions. Sixteen months following treatment, CT studies showed no changes in sizes and appearance of the paravertebral mass (Figure 6). After thirty-nine months, she was readmitted for lung infection and cured by anti-infection therapy.

## 3. Discussion

EMH most commonly develops within the liver and spleen; however, it may also occur in other sites such as the thymus, central nervous system, lymph nodes, lung, pleura, myocardium, kidney, retroperitoneum, and paravertebral areas of thorax with unknown etiology [1]. Extramedullary hematopoietic masses are often microscopic and asymptomatic, but may lead to tumor-like growths. Our initial diagnosis for the patient was tuberculosis with tuberculous pleurisy or lymphoma. However, no tubercle bacillus in the pleural fluid was found. CT and MRI results showed no caseous necrosis, and lymph nodes in a stringy configuration were not consistent with descriptions of lymphoma. Lymph node biopsy revealed acid fast stain negative and disorganized architecture. Immunohistochemistry results suggested the existence of T lymphocytes (CD3, CD43), B lymphocytes (CD20, CD79a), and hematopoietic elements (MPO, CD15) that were consistent with changes in EMH. Due to death during CT-guided puncture in another report [2], we performed the biopsy under direct vision to minimize bleeding.

Primary myelofibrosis (PMF) is a myeloproliferative disorder characterized by bone marrow fibrosis. Blood and bone marrow changes, including leukoerythroblastosis, bone marrow fibrosis, and osteosclerosis, were found on peripheral blood smear and in the bone marrow. The JAK2V617F mutation is a key molecular finding presented in 60% of PMF cases [3]. Since this patient's JAK2V617F mutation was positive and the peripheral blood and bone marrow of the patient were also typical, we arrived at a final diagnosis of PMF. 

Pleural EMH in myelofibrosis has been previously reported. These patients often had no respiratory symptoms, and diagnosis was made at autopsy. However, several patients presented with hemothorax or chylothorax [2, 4, 5]. Mechanisms underlying pleural effusion remain unknown. In most cases or in general, treatment for patients with pleural effusion caused by EMH is often radiotherapy, and some were treated with surgery or an intrathoracic injection of tetracycline or bleomycin [2]. Since radiation might increase symptoms due to tissue edema and intrathoracic tetracycline aggravated hemothorax [4], we used cisplatin and dexamethasone for this patient. With this approach, pleural fluid decreased significantly and was ultimately resolved.

In the past 20 years, new therapies have been developed for the management of PMF; however, patient survival benefits have not significantly changed. Conventional drug therapies for PMF include androgen preparations, danazol, cortico steroids, thalidomide, or lenalidomide and hydroxyurea. Palliative surgery (e.g., splenectomy) or radiation therapy for symptomatic EMH and allogeneic hematopoietic cell transplant (allo-HCT) can be applied in young patients [3]. This patient was treated with cisplatin and dexamethasone, together with prednisone, hydroxyurea, and thalidomide; we herein show that these approaches improve symptoms and attenuate abnormalities in peripheral blood findings with no significant changes in the EMH mass. We believe that these approaches are feasible and show promising efficacy for this patient. However, more studies are needed to further confirm these findings.
